# Squared diffusion-weighted imaging for improving the detection of clinically significant prostate cancer

**DOI:** 10.1038/s41598-025-86068-x

**Published:** 2025-01-27

**Authors:** Birte Valentin, Lars Schimmöller, Matthias Boschheidgen, Tim Ullrich, Thomas Andreas Thiel, Alexandra Ljimani, Jan Philipp Radtke, Thomas Benkert, Peter Albers, Gerald Antoch, Hans-Jörg Wittsack, Michael Quentin

**Affiliations:** 1 Department of Diagnostic and Interventional Radiology, Medical Faculty, University Dusseldorf, Moorenstr. 5, 40225 Dusseldorf, Germany; 2 Department of Diagnostic, Interventional Radiology and Nuclear Medicine, Marien Hospital Herne, University Hospital of the Ruhr-University Bochum, Herne, Germany; 3 Department of Urology, Medical Faculty, University Dusseldorf, Moorenstr. 5, 40225 Dusseldorf, Germany; 4 MR Application Predevelopment, Siemens Healthineers AG, Forchheim, Germany; 5 Center for Integrated Oncology Aachen Bonn Cologne Düsseldorf (CIO ABCD), Düsseldorf, Germany

**Keywords:** Prostate, Prostate cancer, Magnetic resonance imaging

## Abstract

Aim of this study was to proof the concept of optimizing the contrast between prostate cancer (PC) and healthy tissue by DWI post-processing using a quadrature method. DWI post-processing was performed on 30 patients (median age 67 years, prostate specific antigen 8.0 ng/ml) with PC and clear MRI findings (PI-RADS 4 and 5). Multiparametric MRI was performed at 3 Tesla. A multi-shot readout segmentation (rs-EPI) plus zoomed single-shot imaging (z-EPI) sequence was used in 15 patients (group 1) and a single-shot echo-planar imaging (ss-EPI) plus rs-EPI sequence in 15 patients (group 2). B-value images (b1000 and b1800/2000) were squared and then evaluated objectively software-based and subjectively using a 5-point. The squared DWI technique showed a significantly higher contrast ratio (CR) for ss-EPI b1800 (*p* < 0.001), rs-EPI b1000 (*p* < 0.001), rs-EPI b1800 (*p* < 0.001), z-EPI b1000 (*p* = 0.008) and for z-EPI b2000 (*p* < 0.001). After post-processing, a significant improvement in the subjective assessment of image quality was observed for rs-EPI b1000 (*p* < 0.001), rs-EPI b1800 (*p* < 0.001) and z-EPI b1000 (*p* < 0.001). The application of the square post-processing to DWI results in a significant improvement in the CR between PC and healthy tissue, especially at high b-values of ss-EPI or re-EPI. This method may help to improve the detection and differentiation of PC lesions.

**Supplementary Information:**

The online version contains supplementary material available at 10.1038/s41598-025-86068-x.

## Introduction

Multiparametric magnetic resonance imaging (mpMRI) has emerged as a powerful, non-invasive tool for the detection, characterization, and staging of prostate cancer (PC)^1^. According to the Prostate Imaging Reporting and Data System (PI-RADS) recommendations, mpMRI of the prostate comprises an anatomical T2-weighted imaging (T2WI) sequence, a diffusion-weighted imaging (DWI) sequence, and a dynamic contrast-enhanced (DCE) sequence^2,3^. Currently, there is an ongoing debate regarding the potential omission of the DCE in mpMRI of the prostate, resulting in biparametric MRI (bpMRI). This is due to the fact that contemporary contrast agents can cause side effects such as gadolinium deposits or allergic reactions^4^. The PROMIS study suggests that results without DCE can be comparable to those obtained with contrast agents^5,6^. If DCE is omitted, DWI becomes the sole functional sequence for bpMRI of the prostate and therefore gains additional significance.

DWI measures the Brownian motion of water molecules in tissue and provides information on tissue cellularity. Tumor tissue has a higher cellularity and restricted water diffusion compared to benign tissue, resulting in higher signal intensity (SI) on DWI. Furthermore, restricted water diffusion correlates with tumor aggressiveness^7,8^. Conventionally, DWI uses single-shot echo-planar imaging (ss-EPI). However, in recent years, a number of advanced DWI approaches have been developed to improve the accuracy and reliability of PC detection. The most commonly used DWI sequences for mpMRI of the prostate, besides ss-EPI, are zoomed single-shot imaging (z-EPI) with advanced image processing or multi-shot readout segmentation (rs-EPI)^9,10^. Ss-EPI is a valuable and widely used MRI technique, but has several drawbacks that need to be considered. Ss-EPI can be susceptible to image distortion and artifacts, signal loss due to T2* decay, sensitivity to motion, and limitations in its ability to provide anatomical detail. Rs-EPI reduces the effects of motion artifacts and other sources of image distortion. The z-EPI sequence produces high-resolution images with improved contrast and reduced acquisition time. Regardless of the diffusion technique, imaging parameters such as b-values, acquisition time, and slice thickness affect the quality of all diffusion images. High-resolution images are essential for detecting small lesions, while adequate contrast between different tissue types is important for differentiating between PC and non-cancerous tissue. As recommended by PI-RADS v2.1, DWI sequences that mathematically extrapolate a higher b-value image based on the measured b-values are already available at most sites^2,11^. These calculated sequences are intended to further enhance the contrast between different tissue compositions and have the advantage of minimizing acquisition time, making it more comfortable for the patient and cost-effective for the site.

In this study, we used for the first time a novel post-processing squaring method of the DWI sequence, aiming to proof the concept for clearly MRI-visible PC lesions and therefore its potential contribution to the diagnosis of PC in the human prostate. This study serves as a feasibility study to assess the applicability of this post-processing squaring method in the human prostate. Furthermore, the study aims to differentiate whether the contrast between PC tissue and healthy tissue can be optimized by post-processing the DWI sequence using this squaring method. In this context, it was investigated whether the post-processed squared sequences could produce similar contrast-to-noise ratios (CNR) compared to the calculated sequences.

## Materials and methods

### Study design

This retrospective study was approved by the Institutional Review Board and additional informed consent was waived (Clinical ethics committee, KEK, University Hospital Dusseldorf, Heinrich-Heine University, Medical Faculty; Study-No: 5910R; Study ID: 2017034171). No examination was ordered separately for the study. All methods were performed in accordance with the relevant guidelines and regulations and the declaration of Helsinki. Inclusion criteria included examinations with all three sequences are of optimal diagnostic quality (PI-QUAL 5) in the patient’s previous treatment and clear MRI findings (PI-RADS 4 and 5)^12^. Exclusion criteria included a history of prostate surgery, radiotherapy, brachytherapy, and suboptimal image quality (IQ). All thirty included patients underwent prostate mpMRI according to ACR PI-RADS v2.1 criteria^2^. Fifteen consecutive PI-QUAL 5 MRI studies of patients with proven PCA in the peripheral zone (PZ) of the prostate with rs-EPI sequence and z-EPI sequence were included from January 2022 to March 2022. An additional 15 consecutive patients with PI-QUAL 5 and proven PCA with ss-EPI sequence and rs-EPI sequence were included from May 2018 to August 2018. The two different consecutive time points are due to the fact that our current prostate mpMRI protocols include rs-EPI and z-EPI sequences. However, as the ss-EPI sequence is still widely used, we also wanted to include this sequence and therefore had to refer to studies from 2018.

### Imaging protocols

MpMRI was performed at a 3 Tesla MRI scanner (MAGNETOM Prisma, Siemens Healthineers, Erlangen, Germany) using a 60-channel phased-array surface coil. According to the PI-RADSv2.1, all protocols included T1WI, T2WI, DWI, and DCE sequences^2,3^.

*ss-EPI*: We have used a field of view of 200 × 200 mm for the ss-EPI sequence. The b-values were set to 50, 500, 1000 s/mm^2^ and calculated to 1800 s/mm^2^, a slice thickness of 3 mm, a TR of 5300 ms and a TE of 66 ms were used.

*rs-EPI*: A field of view of 200 × 200 mm was used for the rs-EPI sequence. The b-values were set to 0, 1000 s/mm^2^ and calculated to 1800 s/mm^2^, a slice thickness of 3 mm, a TR of 4540 ms and a TE of 50 ms were used.

*z-EPI*: A field of view of 91 × 150 mm was used for the z-EPI research application sequence. The b-values were set to 50, 500, 1000 s/mm^2^ and calculated to 2000 s/mm^2^, a slice thickness of 3 mm, a TR of 3500 ms and a TE of 67 ms were used.

### Objective analysis of image quality

The sequences were exported as DICOM from the local picture archiving and communication system (PACS; Sectra Imtec AB, IDS7, Linköping). The DWI images were squared using specially developed software and normalized to the same mean image intensity as before the squaring was performed. The unprocessed and processed sequences were evaluated using the *syngo.*via software (Siemens Healthineers, Erlangen, Germany). The PC lesions were drawn by a radiologist (B.V. with 5 years of experience) according to the imaging report and histopathological results using the freehand ROI (Supp. Figure 1). To ensure the same ROI position and size in each sequence, the ROI was inserted into each unprocessed and processed sequence using the “Copy to Same Organ Position” function. In addition, round shaped ROIs were placed in healthy reference tissue of the prostate, muscle tissue, and urinary bladder lumen (Supp. Figure 1). Assuming a homogeneous composition of urine in the bladder, the standard deviation (SD) of the respective SI of the urine in the bladder was defined as image noise.

The mean of the SI of each ROI and its SD were documented.

The calculation was performed as follows:

$$CR=\frac{\left(SI\,tumor\,lesion-SI\,healthy\,reference\,prostate\,tissue\right)}{SD\,bladder\,lumen}$$

Abbreviations: CR = contrast ratio, SI = signal intensity, SD = standarddeviation.

### Subjective analysis of image quality

Subjective IQ assessment of all three DWI sequences of high and low b-values with and without square calculation was performed in a blinded consensus by two radiologists (L.S. and B.V., with 12 and 5 years of experience in reading prostate MRI, respectively). Subjective IQ was graded on a 5-point Likert scale from unacceptable^1^, poor^2^, fair^3^, good^4^ to excellent^5^. Criteria included presence of artifacts, delineation of anatomical structures and boundaries, overall sharpness, contrast, and overall subjective impression.

### Statistics

Statistics were performed using SPSS Statistics version 22 (IBM, Chicago, IL, USA) and Microsoft Excel 2016 (Redmond, WA, USA). Statistical differences in CR of each lesion were evaluated using a Mann-Whitney U test with a significance level of 0.05.

## Results

### Patient characteristics

All patient characteristics are listed in Table 1. Two different DWI sequences were acquired per patient. The rs-EPI sequence was obtained for all 30 patients enrolled in the study. In addition, half of the patients received additionally an ss-EPI sequence, while the other half received a z-EPI sequence in addition. This resulted in 30 cases with rs-EPI sequence, 15 with ss-EPI sequence, and 15 with z-EPI sequence for analysis. According to the International Society of Urological Pathology Grade Group (ISUP GG), prostate biopsy of the PC suspicious lesions revealed ISUP GG 1 PC in 1 patient, ISUP GG 2 PC in 12 patients, ISUP GG 3 PC in 9 patients, ISUP GG 4 PC in 4 patients, and ISUP GG 5 PC in 2 patients. Two patients had clear PC lesion (PI-RADS 5) and highly suspicious prostate-specific antigen density, but no histopathology available at the time of study analysis. All PC lesions were located in the peripheral zone, with two patients exhibiting tumor infiltration into the anterior fibromuscular stroma and the transition zone.

**Table 1:** Baseline characteristics and histopathological results of the included patients.

Patients included (*n*)	30
Age (yr), median (IQR)	67 (55–77)
PSA (ng/ml) median (IQR)	8 (5.6–11.1)
Prostate volume (ml), median (IQR)	36 (31–46)
PSAD (ng/ml/ml), median (IQR)	0.22 (0.15–0.31)
PI-QUAL, median (IQR)	5 (5–5)
PI-RADS	
4 (n)	10
5 (n)	20
ISUP GG	
1 (n)	1
2 (n)	12
3 (n)	9
4 (n)	4
5 (n)	2
Biopsy refused (n)	2

PSA = prostate specific antigen; PSAD = prostate specific antigen density; PI-QUAL = Prostate Imaging Quality; PI-RADS = Prostate Imaging Reporting and Data System; IQR = interquartile range; ISUP GG = International Society of Urological Pathology Grading Group.

### Objective IQ analysis

**Fig. 1:**
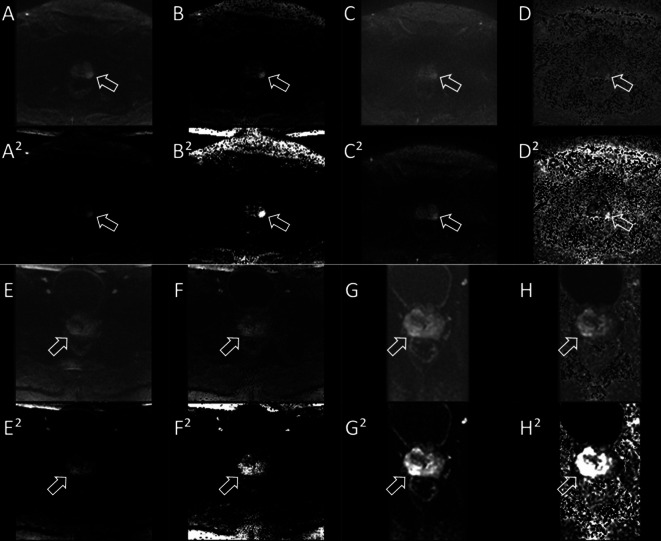
Diffusion sequences of two patients (Patient 1 on top, Patient 2 below). The prostate cancer lesion is marked with an arrow. Presented are the respective sequences [(**A**) rs-EPI b1000, (**B**) rs-EPI b1800, (**C**) ss-EPI b1000, (**D**) ss-EPI b1800, (**E**) rs-EPI b1000, (**F**) rs-EPI b1800, (**G**) z-EPI b1000, (**H**) z-EPI b2000] and the squared technique [(**A**^**2**^)-(**H**^**2**^)]. Except for the ss-EPI b1000 sequence, the CR was significantly enhanced in all other sequences.

**Fig. 2:**
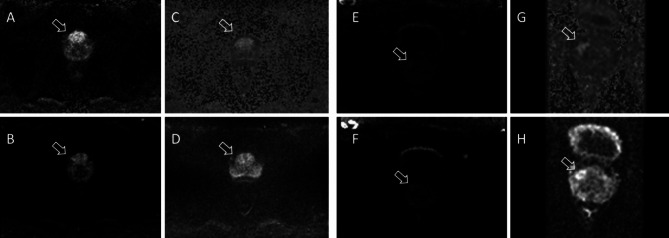
Comparison of all examined sequence types [(**A**) rs-EPI calc 1800, (**B**) rs-EPI b1000 squared, (**C**) ss-EPI calc b1800, (**D**) ss-EPI b1000 squared, (**E**) z-EPI calc b2000, (**F**) z-EPI b1000 squared] between the calculated b-values (**A**, **C**, **E**) and the squared (**B**, **D**, **F**) b-values of the respective sequence. With both variants, a similarly good contrast can be achieved.

Squaring the DWI resulted in significantly higher CR values for all sequences, except for the ss-EPI uncalculated sequence with a b-value of 1000 s/mm^2^ (see Table 2; Fig. 1). When comparing the unsquared calculated sequences with the squared uncalculated sequences, a significantly higher CR was observed for the unsquared calculated sequences (z-EPI: *p* = 0.026, ss-EPI: *p* = 0.002, rs-EPI: *p* = 0.009; see Table 3; Fig. 2).

**Table 2:** Contrast ratio (CR) as the percentage difference between the original and the processed technique.

Sequence	Absolute CR difference absolut [%]	*P* value
ss-EPI b1000	232.21	0.367
ss-EPI b1800	489.31	0.001
rs-EPI b1000	251.47	< 0.001
rs-EPI b1800	205.21	< 0.001
z-EPI b1000	193.37	0.008
z-EPI b2000	884.51	< 0.001

CR = contrast ratio; ss-EPI = single shot echo planar imaging (EPI) sequence; rs-EPI = multi shot readout segmented EPI sequence; z-EPI = zoomed single shot EPI.

**Table 3:** Contrast ratio (CR) values as a total and as a percentage difference between these sequences.

Sequence	Absolut CR difference (%)	CR difference in multiples	*P* value
ss-EPI (*n* = 15)	357.18	5.94	0.002
rs-EPI (*n* = 30)	187.18	6.85	0.009
z-EPI (*n* = 15)	180.93	12.76	0.026

CR = contrast ratio; ss-EPI = single shot echo planar imaging (EPI) sequence; rs-EPI = multi shot readout segmented EPI sequence; z-EPI = zoomed single shot EPI.

### Subjective IQ Analysis

**Fig. 3:**
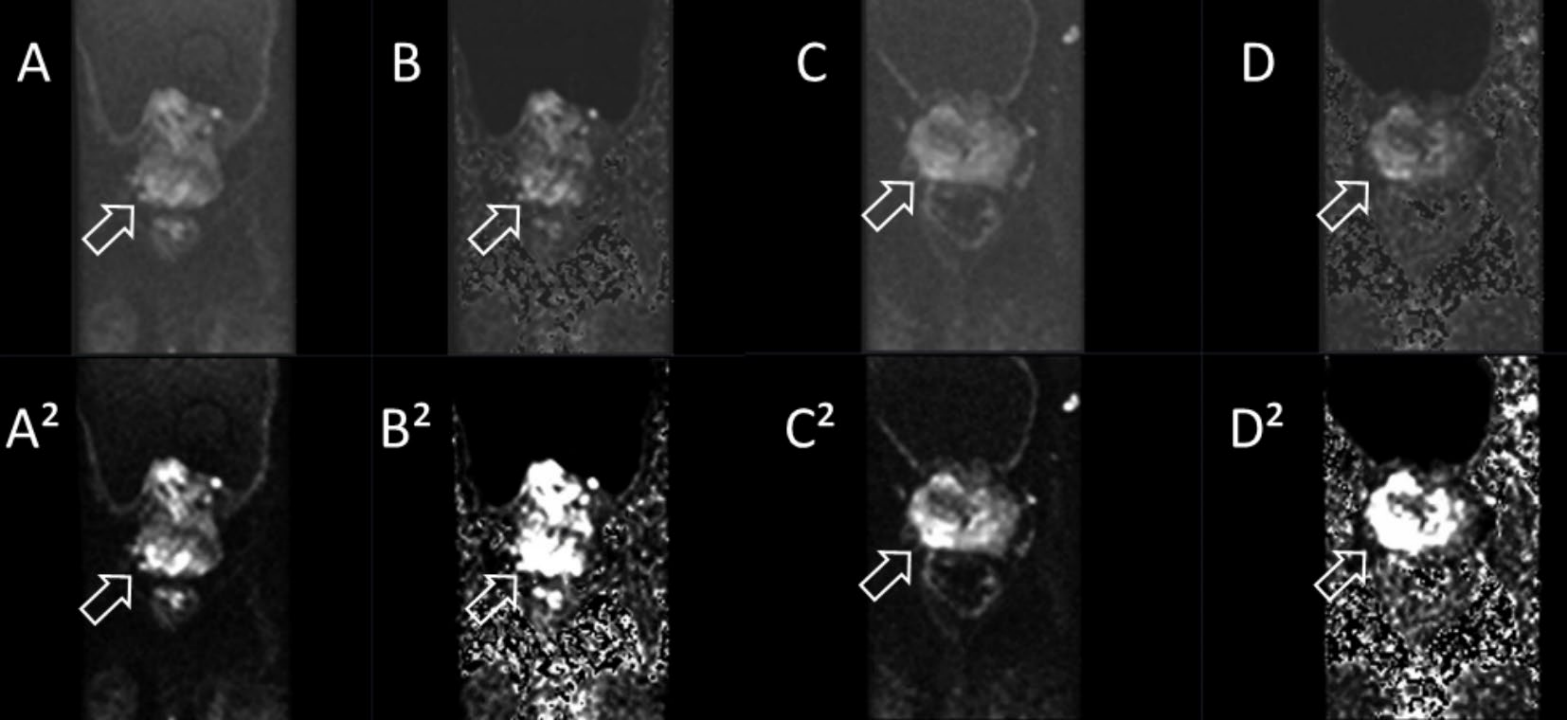
Overestimation of the tumor margins upon squaring the high calculated b-values (**B**^**2**^, **D**^**2**^). Shown are respective sequences of a 58 year old patient with a T3b prostate cancer. The prostate cancer lesion is marked with an arrow. (**A**) rs-EPI b1000, (**B**) rs-EPI b1800, (**C**) z-EPI b1000, (**D**) z-EPI b2000 and the squared sequences (**A**^**2**^-**D**^**2**^).

Table 4 presents the results of the subjective IQ analysis, showing a significant improvement in subjective IQ for almost all sequences. However, squaring the low and high b-value ss-EPI sequences and the high b-value z-EPI sequence did not result in a significant improvement. Blooming at the edges of lesions was observed; primarily on high b-value images (see Fig. 3).

**Table 4:** Subjective image quality ratings of the original and processed DWI technique.

Subjective IQ	Original DWI, median (IQR)	Processed DWI, median (IQR)	*P*-value
ss-EPI b1000	2 (1–2)	3 (2–3)	0.089
ss-EPI b1800	3 (2–4)	4 (2–5)	0.106
rs-EPI b1000	2 (2–3)	3 (3–4)	< 0.001
rs-EPI b1800	4 (3–4)	5 (4–5)	< 0.001
z-EPI b1000	3 (3–3)	5 (4–5)	< 0.001
z-EPI b2000	4 (4–5)	4 (3–5)	0.436

IQ = image quality; ss-EPI = single shot echo planar imaging (EPI) sequence; rs-EPI = multi shot readout segmented EPI sequence; z-EPI = zoomed single shot EPI; IQR = interquartile range.

## Discussion

We have successfully implemented a post-processing squaring method for DWI in a human prostate. The squaring method improved the CR between PC tissue and healthy tissue and maybe used as an alternative to the acquisition of additional high b-value images to improve the detection of PC lesions. This could be particularly beneficial for bpMRI for patients who are either unable or unwilling to receive contrast agents to further improve DWI accuracy.

With the growing evidence for bpMRI, the continued improvement of the only remaining functional DWI sequence becomes even more important^13^. In the past, several groups have investigated and evaluated different DWI sequences for their accuracy and usefulness in detecting PC lesions^13^. Studies by Klingebiel et al. and Thierfelder et al. found z-EPI to be a superior DWI sequence compared to ss-EPI and partly also for rs-EPI sequence^9,14^. This observation was also confirmed in our cohort. The z-EPI sequence showed an improved CR compared to the ss-EPI and rs-EPI sequences, both when comparing uncalculated and calculated baseline values with their respective processed squared sequences. The improved clarity in identifying a PC lesion on DWI, characterized by post-processing increased DWI SI, may have the potential to upgrade bp PI-RADS score for DWI from 3 to 4 without the need for contrast agent application. Another key advantage of this post-processing technique is its ability to preserve MRI capacity by not affecting the duration of the MRI examination, thus optimizing diagnostic outcomes.

Our results show that, despite squaring, the lower b-values show less enhancement than the higher b-values. However, it is worth considering whether further processing of the already calculated sequences is advisable. As these sequences are based on the extrapolation of the measured b-values, additional processing could potentially amplify any existing errors, affecting image interpretation and potentially leading to false positive results. The processed sequences of the high and low b-values of the rs-EPI and the low b-values of the z-EPI showed a significant improvement in subjective IQ compared to the unprocessed sequences. This contrasts with the high b-values for the ss-EPI sequence, as a significant improvement in CR was seen with this sequence. Although the CR was significantly higher for the high b-values of the processed b2000 z-EPI sequence, the subjective IQ analysis showed a poorer delineation of the lesion boundaries as well as an increased overexpression of the SI of the remaining tissue, resulting in a reduced overall sharpness and a less favorable overall subjective impression. Therefore, in our opinion, squaring of sequences up to a b-value of 1800 s/mm^2^ is beneficial, but not recommended for the very high b-values, such as b = 2000 s/mm^2^.

One of the advantages of mpMRI is its ability to provide valuable pre-biopsy information regarding the aggressiveness of PC^7^. The processing of very high b-value sequences can lead to overexposure of the lesion and over enhancement of non-specific DWI restrictions. This overexposure can lead to misinterpretation of tumor margins, resulting in an overestimation of tumor lesion size. This limited correlation of the DWI sequence with tumor size has already been described by Rosenkrantz et al.^15^. Therefore, it is crucial to include a variety of sequence types in the assessment of prostate lesions, as advocated by PI-RADS v2.1. We recommend the mapping of potential tumor lesions for biopsy targets based on the low ADC signal in the anatomical T2 sequences.

There are some limitations that need to be addressed. This retrospective study, involving a limited number of patients with clear MRI visible PC lesions, aims to evaluate the applicability of the post-processing squaring method in the human prostate. We only included PI-QUAL 5 examinations and PI-RADS 4 and 5 cases. Thus, the sequences were of optimal diagnostic quality and artifacts such as folding, motion artifacts or artifacts due to rectal gas did not affect the post-processing method. As some patients in clinical routine have e.g. a hip prosthesis or cannot take spasmolytics due to contraindications, these artifacts may have an effect on the post-processing. Also, more unclear cases (e.g., PI-RADS 3) were not addressed in this evaluation. Nevertheless, we were able to demonstrate that the post-processing squaring method can significantly increase the objective and subjective IQ of a clear PC lesion.

In conclusion, the proposed post-processing squaring approach results in improved CR between PC lesions and healthy tissue. This method can provide an optimization of CR and thus an improvement in tumor detection without relevant time investment. Considering the increasing preference for bpMRI, this easily applicable post-processing method can improve the diagnosis of PC lesions. Especially in the case of a peripheral PI-RADS 3 lesion, this post-processing method could favour an upgrade or downgrade to a PI-RADS 4 or PI-RADS 2 lesion in the case of bpMRI. Also, the squared technique might be an alternative to replace an additional high b-value assessment if automatic calculation is not feasible. This needs to be analyzed in a larger study.

## Electronic supplementary material

Below is the link to the electronic supplementary material.

Supplementary Material 1

Supplementary Material 2

## Data Availability

The datasets used and/or analyzed during the current study available from the corresponding author on reasonable request.
