# Supplementary material for: Squared diffusion-weighted imaging for improving the detection of clinically significant prostate cancer
Source: Sci Rep. 2025 Jan 27;15:3451. doi: 10.1038/s41598-025-86068-x (PMC11772849; doi:10.1038/s41598-025-86068-x)
Supplement: Supplementary file 1 — Supplementary Material 1 [file 41598_2025_86068_MOESM1_ESM.docx]

**Supp. Table 1:** Sequence parameters of rs-EPI, z-EPI, and ss-EPI.

| **DWI sequence parameters** | **rs-EPI** | **z-EPI** | **ss-EPI** |
| --- | --- | --- | --- |
| **MR scanner** | 3 Tesla MAGNETOM Prisma, Siemens Healthineers | | |
| **Coil** | 60-channel phased-array surface coil | | |
| **TR (ms)/ TE (ms)** | 5450/80 | 3500/67 | 5300/66 |
| **b-values (s/mm^2^)** | 0, 1000 | 50, 500, 1000 | 50, 500, 1000 |
| **Calculated**  **b-values (s/mm^2^)** | 1800 | 2000 | 1800 |
| **Voxel size (mm)** | 1.4 x 1.4 x 3.0 | 0.9 x 0.9 x 3.0 | 1.5 x 1.5 x 3.0 |
| **Field of View (mm)** | 200 x 200 | 89 x 150 | 200 x 200 |
| **Matrix (mm)** | 140 | 82 | 140 |
| **Number of averages** | 1, 3 | 2, 4, 9 | 3, 5, 10 |
| **Number of slices** | 32 | 32 | 32 |
| **Acquisition Time (min:s)** | 6:38 | 3:35 | 4:03 |

DWI = diffusion weighted imaging; EPI = echo planar imaging; TR = time of repetition; TE = time of echo; ss-EPI = single shot EPI sequence; rs-EPI = multi shot readout segmented EPI sequence; z-EPI = zoomed single shot EPI
