# Supplementary figures and images for: Squared diffusion-weighted imaging for improving the detection of clinically significant prostate cancer
Source: Sci Rep. 2025 Jan 27;15:3451. doi: 10.1038/s41598-025-86068-x (PMC11772849; doi:10.1038/s41598-025-86068-x)

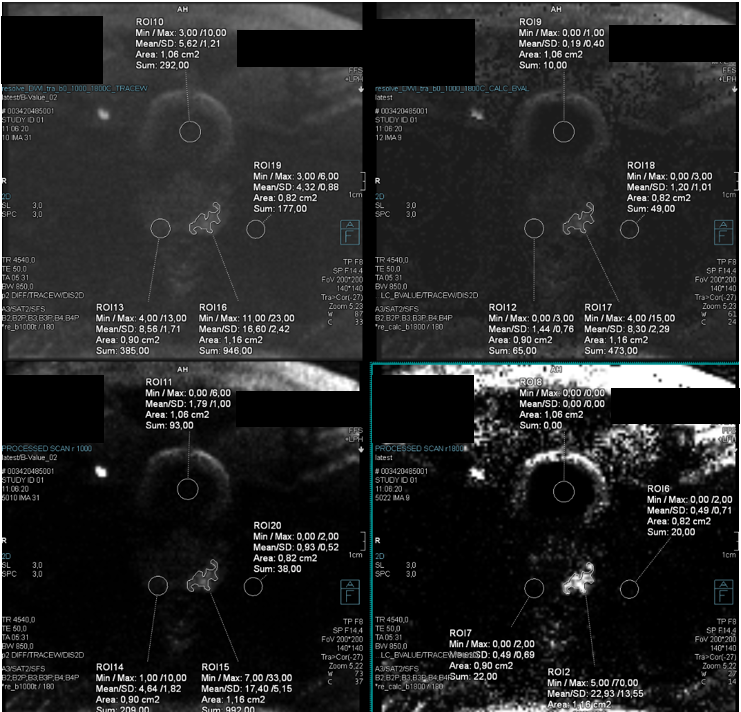
**Supp. Figure 1:** ROI placement for objective analysis of image quality.

Supplement: Supplementary file 2 — Supplementary Material 2 [file 41598_2025_86068_MOESM2_ESM.docx]
